# Spatial Distribution of COVID-19 Hospitalizations and Associated Risk Factors in Health Insurance Data Using Bayesian Spatial Modelling

**DOI:** 10.3390/ijerph20054375

**Published:** 2023-02-28

**Authors:** Boris Kauhl, Jörg König, Sandra Wolf

**Affiliations:** 1 AOK Nordost—Die Gesundheitskasse, Brandenburger Str. 72, 14467 Potsdam, Germany; 2 Institute for Health Services Research in Dermatology and Nursing (IVDP), University Medical Center Hamburg-Eppendorf (UKE), Martinistraße 52, 20246 Hamburg, Germany

**Keywords:** COVID-19, GIS, health insurance, Bayesian spatial modelling

## Abstract

The onset of COVID-19 across the world has elevated interest in geographic information systems (GIS) for pandemic management. In Germany, however, most spatial analyses remain at the relatively coarse level of counties. In this study, we explored the spatial distribution of COVID-19 hospitalizations in health insurance data of the AOK Nordost health insurance. Additionally, we explored sociodemographic and pre-existing medical conditions associated with hospitalizations for COVID-19. Our results clearly show strong spatial dynamics of COVID-19 hospitalizations. The main risk factors for hospitalization were male sex, being unemployed, foreign citizenship, and living in a nursing home. The main pre-existing diseases associated with hospitalization were certain infectious and parasitic diseases, diseases of the blood and blood-forming organs, endocrine, nutritional and metabolic diseases, diseases of the nervous system, diseases of the circulatory system, diseases of the respiratory system, diseases of the genitourinary and symptoms, and signs and findings not classified elsewhere.

## 1. Introduction

The COVID-19 pandemic has already and still continues to impact billions of people across the world and has been declared a public health emergency of international concern by the World Health Organization (WHO) [1]. To contain the spread of the virus, lockdowns across the globe were declared, resulting in closure of cities, suspension of schools, and restrictions of international travel, resulting not only in a public health crisis, but also in a humanitarian, economic and social crisis [2,3].

In Germany, the first case was reported in Bavaria at the end of January 2020. At the beginning of March, almost all federal states in Germany reported cases of the disease. The southern counties in Bavaria and Baden-Württemberg in particular were affected by high numbers of cases [4]. From March 16, the first lockdown was imposed: far-reaching exit and contact restrictions applied, which were only gradually lifted again at the end of April.

Several studies from German-speaking countries have investigated the spread of COVID-19 infections from a spatiotemporal perspective. The first study [5] dates from May 2020 and is known as the Ischgl study. Based on a spatial diffusion model, correlations between the occurrence of COVID-19 infections in Germany and population mobility could be established for the first time. The vacation resort of Ischgl in Austria was given special importance as a starting point for infection occurrence in Germany, which primarily brought into focus the importance of mobility as a driver of virus spread. Steiger et al., in their study on the determinants of regional infection incidence at the level of districts and district-free cities in the period from 15 February 8 to July 2020, found that increasing temperature and mobility for basic supplies, especially, reduce the incidence of infection, whereas recreational mobility or precipitation can increase the incidence of infection [6]. In their study, Scarpone et al., analysed spatial associations between COVID-19 case rates and spatial characteristics of infrastructure, sociodemographics, and the built environment [7]. In summary, the results showed, among others, an association between built density, place of residence, transportation infrastructure (e.g., access to intensive care units), and sociodemographic factors (e.g., unemployment) as predictors of regional incidence rates in Germany.

Overall, it is clear that mobility and sociodemographic circumstances in particular have an important influence on the regional incidence of infection. In addition, it has been shown that density, built-up areas, and even weather influence the frequency of contact. Importantly, the determinants overlap spatially and temporally [8] and also depend on the pandemic phase [9]. For example, in the early pandemic phase until mid-April 2020, a socioeconomic gradient with higher incidence in less deprived regions of Germany is evident, but this gradient dissipates or reverses in favour of more deprived regions in the south of the country as the pandemic progresses [10]. This highlights the need to consider spatiotemporal dynamics within the observation period when analysing COVID-19 determinants with infection incidence, as the predictors of incidence rates are spatiotemporally dependent on the pandemic phase.

The fast spread of COVID-19 has increased public awareness of the use of geographic information systems (GIS) for pandemic preparedness, resulting in a large number of studies revealing the potential of GIS and spatial statistics—especially cluster detection methods—to detect outbreaks [3,11,12,13]. Likewise, GIS has also been extensively used to identify sociodemographic and environmental characteristics associated with COVID-19, possibly resulting in a better understanding of the population groups most at risk [14,15].

In Germany, most research on the spatial distribution of COVID-19 is restricted to the relatively coarse level of counties [16], masking important variation at the small-area, municipality, or even neighbourhood level, hampering productive outbreak detection and management, despite numerous studies’ having shown the value of microgeographic data on COVID-19 [17,18,19].

Likewise, studies on the spatiotemporal dynamics focus mainly on cluster detection methods, with SaTScan (Software for the spatial, temporal, and space–time scan statistics) being the most widely used statistics software [19,20]. Cluster tests are an important tool here to effectively detect outbreaks.

A large number of studies examined sociodemographic risk factors for COVID-19. However, the majority of studies are based on an ecological study design and not at the individual level [14,15]. While these studies have the advantage in that they may represent the total population, they suffer from ecological fallacy, meaning that the results of a study design based on aggregated data do not necessarily represent associations at the individual level. In contrast, studies based on individual data often suffer from small population samples (e.g., a hospital) [21,22].

In this context, health insurance data might not only provide fairly detailed insights into the spatial and spatiotemporal distribution, since these data can be analysed at microgeographic level, but also provide a rich and detailed data source on individual-level sociodemographic information and pre-existing medical conditions.

The aim of this research is therefore to (i) provide insight into the spatial distribution of COVID-19 hospitalizations based on the data of northeast Germany’s largest statutory health insurance provider and (ii) analyse sociodemographic and medical conditions associated with hospitalization.

## 2. Methods

### 2.1. Data

AOK Nordost is the largest statutory health insurance provider in northeast Germany and covers approximately 25% of the population in the three federal states of Berlin, Brandenburg, and Mecklenburg-Western-Pomerania.

For this study, we used all 1.7 million insurants that were insured in 2021. We defined COVID-19 hospitalization as an insurant having a positive PCR test in a hospital, coded with the international classification of disease (ICD-10) U07.1!. To ensure that we captured only hospitalizations where COVID-19 is likely the primary reason for hospitalization, we additionally restricted our data source to include only individuals that have in addition to U07.1! a diagnosis for viral pneumonia or respiratory syndrome as defined by the ICD-10 codes J12.8, J12.9, J20.8, J20.9, J21.8, J21.9, J22.-. In total, 8402 insurants were hospitalized due to COVID-19.

For the analysis of possible risk factors for COVID-19 hospitalizations, we included sex, age, being unemployed at 1 July 2021, and foreign citizenship. To account for underlying chronic diseases, we included information on whether the insurant had a confirmed diagnosis of diseases, aggregated to ICD-10 chapters to keep the number of possible diagnoses per insurant at a reasonable number. The included ICD-10 chapters consist of I: Certain infectious and parasitic diseases, II: Neoplasms, III: Diseases of the blood and blood-forming organs and certain disorders involving the immune mechanism, IV: Endocrine, nutritional and metabolic diseases, V: Mental and behavioural disorders, VI: Diseases of the nervous system, VII: Diseases of the eye and adnexa, VIII: Diseases of the ear and mastoid process, IX: Diseases of the circulatory system, X: Diseases of the respiratory system, XI: Diseases of the digestive system, XII: Diseases of the skin and subcutaneous tissue, XIII: Diseases of the musculoskeletal system and connective tissue, XIV: Diseases of the genitourinary system, XV: Pregnancy, childbirth and the puerperium, XVI: Certain conditions originating in the perinatal period, XVII: Congenital malformations, deformations and chromosomal abnormalities, and XVIII: Symptoms, signs and abnormal clinical and laboratory findings, not elsewhere classified.

At the aggregated level, we used a commercial dataset from WIgeoGIS of the so-called Geomarkets. A Geomarket is an administrative unit of approximately 300 households and contains valuable information on demographics, socioeconomic information, and household composition of the respective population. This data source is more useful than free official administrative data, which are only available at the level of municipalities, where large cities such as Germany’s capital, Berlin, represent only one single municipality. In contrast, Geomarkets allow an analysis of intra-urban differences. In total, northeast Germany consists of approximately 16,400 Geomarkets. The insurants were aggregated to the level of Geomarkets based on their respective address coordinates. Several studies demonstrated that area deprivation has a significant impact on COVID-19 [23,24]. We therefore calculated a deprivation index based on the following variables: unemployment rate, proportion of employed persons at the place of residence, purchasing power, persons with high school degrees, and proportion of persons without formal education. The domains of employment, income, and education were weighted equally. The resulting index values range from 1 (least deprived) to 100 (most deprived). The methodology is similar to the calculation of the German index of multiple deprivation by Werner Maier [25].

### 2.2. Statistical Analysis

To visualize the cumulative one-year COVID-19 incidence, we aggregated the insurants to the level of the 16,400 Geomarkets based on their address coordinates. To be able to visualize regional differences at this fine level, we used the Besag–York–Mollie (BYM) model. The BYM model has been extensively used to display disease rates at fine spatial resolution [26]. The input for this model consisted of the sex- and age-adjusted number of hospitalized COVID-19 patients and the expected cases. The basic assumption is that the COVID-19 hospitalizations follow a Poisson distribution, where the expected cases are the global average of the sum of observed cases divided by the global sum of insurants, multiplied by the insurants of each Geomarket. The model adjusts for the uneven distribution of the AOK Nordost insurants by weighting the incidence of a Geomarket by the average of the neighbouring Geomarkets and additionally shrinking the rate towards the global mean. This is performed by providing a neighbourhood matrix of the Geomarkets. We chose queen contiguity, where all Geomarkets are defined as neighbours if they share a common edge or border [27]. The model then smooths out the noise associated with small numbers of COVID-19 hospitalization cases as a function of the data in surrounding areas. A more detailed statistical explanation is given by Lawson et al., 2000 [28]. Additionally, we created a continuous surface to preserve insurant confidentiality, by applying an interpolation method called the stochastic partial differential equation (SPDE) approach. This approach has also been used to create small-area continuous surfaces for several diseases such as HIV prevalence in sub-Saharan Africa [29] or disease management enrolment in Germany [30]. The calculation of the BYM model and the SPDE approach was carried out using the integrated nested Laplace approximation available in the INLA package for R version 4 [31], and the results were then displayed with the R package ggplot2 [32].

### 2.3. Regression Analysis

To calculate possible risk factors for COVID-19 hospitalizations, we used a Bayesian global logistic regression model, using the BYM model to account for spatial relationships in the form of structured and unstructured effects at the level of the 16,400 Geomarkets [30,31]. At the individual level, we used sex, age, foreign citizenship, being unemployed at 1 July 2021, and being in a nursing home. At the aggregated level, we used our deprivation index and average household size. We transformed the deprivation index into quintiles and included the index as categories, where the first quintile—the lowest level of deprivation—is the reference category. The response variable was coded as a binary variable (the insurant was hospitalized for COVID-19 vs. was not hospitalized). The regression coefficients were then exponentiated to allow an interpretation as odds ratios, which are easier to interpret than the plain regression coefficients [33,34].

To check for multicollinearity among the explanatory variables, we started with a non-spatial global regression model and checked for multicollinearity using the HH package in R. The HH package assigns a variance inflation factor (VIF) to all explanatory variables within the regression model. A VIF > 5 indicates the presence of multicollinearity and warrants the removal of one or more of the explanatory variables [35].

## 3. Results

### 3.1. Spatial Distribution of Accumulated COVID-19 Incidence 2021

**Figure 1:**
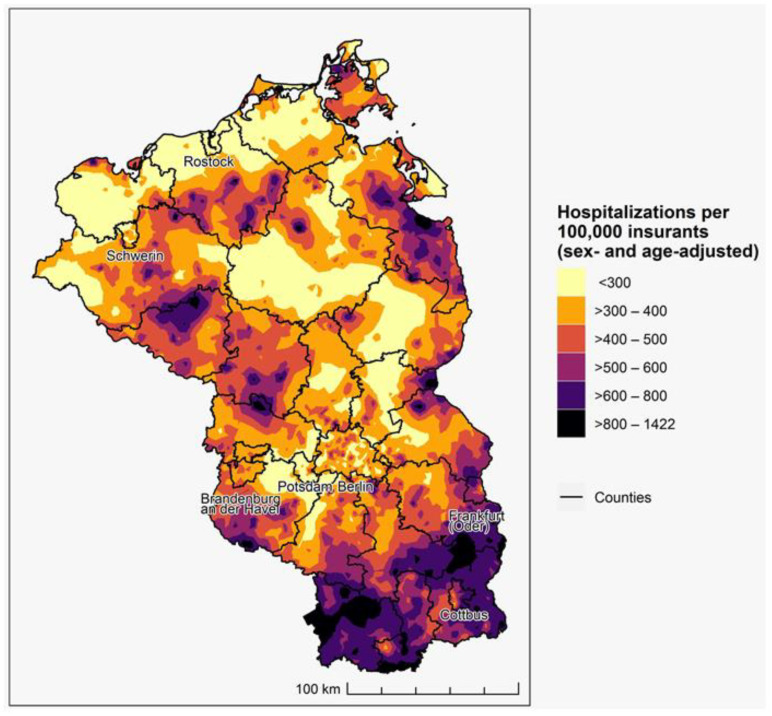
Accumulated one-year incidence of COVID-19 hospitalizations (2021).

The accumulated one-year incidence of COVID-19 hospitalizations ranged between 0 and 1422 hospitalized insurants per 100,000 insurants. The highest incidence could be observed in the south of Brandenburg in the counties of Elbe-Elster and Spree-Neiße, but also in smaller spots scattered across the whole study area (Figure 1). The lowest incidence could be observed on the coastline of Mecklenburg-Western-Pomerania, including the city of Rostock.

### 3.2. Risk Factors for COVID-19 Hospitalizations

Male insurants had a 67.7% higher risk of hospitalizations than women (Table 1). With every year of age, the risk of hospitalization increased by 3.9%. Insurants with foreign citizenship had a 150.2% higher risk than insurants with German citizenship. Being currently unemployed increased the risk by 29.6%. Insurants living in a nursing home had a 75.9% higher risk than insurants not living in a nursing home.

Pre-existing chronic conditions significantly associated with hospitalizations were certain infectious and parasitic diseases, where insurants with this disease group had a 23.6% higher risk. Diseases of the blood and blood-forming organs increased the risk by 29.3%. Endocrine, nutritional and metabolic diseases increased the risk by 35.5%. Diseases of the nervous system increased the risk by 28.4%. Diseases of the circulatory system increased the risk by 21.4%. Diseases of the respiratory system increased the risk by 23.2%. Diseases of the genitourinary system increased the risk by 24.5%. Symptoms, signs and findings not elsewhere classified increased the risk by 16.2%. Average household size did not have a significant impact on the risk of hospitalization. The effect of deprivation was not linear. Only the second-least-deprived quintile and the medium-deprived quintile had a significant effect on the risk of hospitalization: Insurants living in second-least-deprived Geomarkets had an 11% higher risk than insurants living in the least deprived quintile, and insurants living in the medium-deprived quintile had an 8% higher risk than insurants living in the least deprived quintile.

## 4. Discussion

This is likely one of the most spatially detailed research studies in Germany based on health insurance data on COVID-19 hospitalizations.

We found strong spatial differences. The main sociodemographic risk factors for COVID-19 hospitalizations were male sex, higher age, being unemployed, and living in a nursing home. Pre-existing conditions associated with hospitalization were certain infectious and parasitic diseases, diseases of the blood and blood-forming organs, endocrine, nutritional and metabolic diseases, diseases of the nervous system, diseases of the circulatory system, diseases of the respiratory system, diseases of the genitourinary system, and symptoms, signs and findings not elsewhere classified.

Our results clearly demonstrate the benefits of small-area data on COVID-19 hospitalizations. We aggregated the insurants for the accumulated one-year incidence of 2021 to the level of the 16,400 Geomarkets of our study area, which is more detailed by far than the counties, for which official data of the Robert Koch Institute is reported [16,36].

Individual lower socioeconomic status was a risk factor for hospitalization. This is in line with other studies, not only in the German context [37], but in international studies [38]. Our study examined both lower socioeconomic status both at the individual level and at the aggregated level in the form of deprivation at the place of residence at a very detailed spatial resolution. However, we found that mainly individual-level socioeconomic status is a risk factor, but not necessarily living in the least deprived areas.

Similarly, our results confirm that foreign citizenship seems to be a risk factor for more severe consequences from a COVID-19 infection. This has been observed in Germany [39] as well as in other high-income countries [40].

We identified insurants living in nursing homes as another sociodemographic high-risk group. This is not surprising, as persons living in nursing homes generally are fairly old and have a higher number of chronic diseases than average. Logically, these findings are in line with other studies in Germany [41].

While the international literature suggests that area deprivation has an important effect on COVID-19 hospitalization risk [42], we found that insurants living in the second-least and medium-deprived Geomarkets had a higher risk than in the least-deprived Geomarkets. Since our study is based at the microgeographic level of the Geomarkets, this might further reflect the need for more spatially detailed research on COVID-19, as the problem of ecological fallacy grows with the size of the geographical unit for which the data are available [43]. Based on our findings, we might conclude that, at least for our subsample of the population, individual-level socioeconomic status might be more relevant than the place where the insurants live. Since our study included both individual-level socioeconomic status and area-level socioeconomic status, our findings add more depth than previous studies, which mostly included only one measure of socioeconomic status, but seldom both.

## 5. Limitations

Our study has several limitations:

1. The database of AOK Nordost does not contain any information on vaccination status of its insurants. Logically, the positive effect of vaccination could not be quantified. It would have been interesting to quantify the effect of vaccination with regards to date of vaccination, number of doses, and pre-existing conditions on COVID-19 hospitalizations. Such an approach could help to determine in which groups with specific underlying medical conditions vaccination is more effective than in others.
2. Although as cases we selected only those persons who have a laboratory-confirmed diagnosis of COVID-19 as the primary code in addition to a secondary diagnosis of viral pneumonia or respiratory syndrome, it is not clear how high the quality of diagnosis actually is, e.g., COVID-19 being detected as a by-product of another reason for hospital admission.
3. AOK Nordost is northeast Germany‘s largest health insurance provider, covering appr. 25% of the inhabitants. However, large sociodemographic differences of members of different health insurance providers exist, with the AOK Nordost having a higher proportion of elderly and chronically ill persons. As a result, our analysis may not be representative of the whole population. While the prevalence rates may be slightly higher than for all statutory health insurants, the regional distribution of diseases is generally comparable to those of all statutory health insurants [26,44,45,46]. As a result, the general distribution of COVID-19 hospitalizations may be slightly higher than for all statutory health insurants, but the regional distribution is expected to still be comparable.

Additionally, with tests for COVID-19 in 2020 and 2021 having been mostly performed at testing sites and not within ambulatory care, we could not see whether a COVID-19 diagnosis was existent before the insurant was hospitalized. This might influence the validity of our results, since our database contains only hospital diagnoses for COVID-19 for those years.

## 6. Conclusions

This is likely one of the most spatially detailed studies on the spatial distribution of COVID-19 hospitalizations and its associated risk factors. We found important regional variations at very fine scales, clearly demonstrating the need for more fine-grained spatial data on possible future pandemics. Our results clearly identified persons with lower socioeconomic status and persons living in nursing homes as important sociodemographic risk groups. Additionally, we identified several disease groups as risk factors for hospitalizations. COVID-19 hospitalizations and associated risk factors have significant policy implications that must be taken into consideration when creating and implementing mitigation and containment strategies. Age, underlying health conditions, and socio-economic status have been identified as key risk factors for severe illness and hospitalization from COVID-19. Therefore, policies that target vulnerable populations, such as elderly individuals and those with underlying health conditions, are crucial in reducing hospitalizations and deaths from the virus. Additionally, policies that address socio-economic disparities, such as increasing access to healthcare and providing financial support for those who have been impacted by the pandemic, can also have a meaningful impact on reducing hospitalizations. These results might serve as a foundation for better outbreak and containment strategies.

## Figures and Tables

**Table 1:** Regression coefficients for COVID-19 hospitalizations.

Variable	Coefficient	2.5% CI	97.5% CI
Intercept	0.000	0.000	0.000
Sex: male (Ref. female)	1.677	1.603	1.754
Age (in years)	1.039	1.037	1.041
Foreign citizensip (Ref. German)	2.502	2.340	2.675
Unemployed (Ref. not unemployed)	1.296	1.196	1.404
Nursing home (Ref. not in nursing home)	1.759	1.634	1.893
I Certain infectious and parasitic diseases	1.236	1.165	1.311
II Neoplasms	0.964	0.913	1.016
III Diseases of the blood and blood-forming organs and certain disorders involving the immune mechanism	1.293	1.224	1.368
IV Endocrine, nutritional and metabolic diseases	1.355	1.269	1.449
V Mental and behavioural disorders	1.007	0.959	1.058
VI Diseases of the nervous system	1.284	1.223	1.350
VII Diseases of the eye and adnexa	0.947	0.900	0.997
VIII Diseases of the ear and mastoid process	0.942	0.890	0.997
IX Diseases of the circulatory system	1.214	1.124	1.313
X Diseases of the respiratory system	1.232	1.175	1.293
XI Diseases of the digestive system	1.012	0.963	1.063
XII Diseases of the skin and subcutaneous tissue	1.033	0.979	1.089
XIII Diseases of the musculoskeletal system and connective tissue	1.029	0.974	1.088
XIV Diseases of the genitourinary system	1.245	1.182	1.311
XV Pregnancy, childbirth and the puerperium	1.065	0.772	1.468
XVI Certain conditions originating in the perinatal period	1.177	0.689	2.008
XVII Congenital malformations, deformations and chromosomal abnormalities	0.950	0.887	1.019
XVIII Symptoms, signs and abnormal clinical and laboratory findings, not elsewhere classified	1.162	1.097	1.230
Household size	1.058	0.984	1.135
Deprivation 2nd quintile (Ref. 1st quintile)	1.110	1.018	1.208
Deprivation 3rd quintile (Ref. 1st quintile)	1.080	0.989	1.178
Deprivation 4th quintile (Ref. 1st quintile)	1.026	0.932	1.130
Deprivation 5th quintile (Ref. 1st quintile)	1.058	0.984	1.135

CI: Confidence interval.

## Data Availability

The data used in this study contain sensitive information of a health insurance provider (social data). Social data are part of social secrecy (§ 35 SGB I) and have to be kept secret by federal law (§ 78 SGB X). The data may therefore not be made available to third parties.

## References

[1] Sohrabi C., Alsafi Z., O’Neill N., Khan M., Kerwan A., Al-Jabir A., Iosifidis C., Agha R. (2020). World Health Organization declares global emergency: A review of the 2019 novel coronavirus (COVID-19). Int. J. Surg..

[2] United Nations UN Response to COVID-19. https://www.un.org/en/coronavirus/UN-response.

[3] Aral N., Bakir H. (2022). Spatiotemporal Analysis of Covid-19 in Turkey. Sustain. Cities Soc..

[4] Ärzteblatt.de Rückblick 2020: Die Welt im Griff des Virus. https://www.aerzteblatt.de/nachrichten/119821/Rueckblick-2020-Die-Welt-im-Griff-des-Virus.

[5] Felbermayr G., Hinz J., Chowdhry S. (2021). Après-ski: The spread of coronavirus from Ischgl through Germany. Ger. Econ. Rev..

[6] Steiger E., Mussgnug T., Kroll L.E. (2021). Causal graph analysis of COVID-19 observational data in German districts reveals effects of determining factors on reported case numbers. PLoS ONE.

[7] Scarpone C., Brinkmann S.T., Große T., Sonnenwald D., Fuchs M., Walker B.B. (2020). A multimethod approach for county-scale geospatial analysis of emerging infectious diseases: A cross-sectional case study of COVID-19 incidence in Germany. Int. J. Health Geogr..

[8] Kuebart A., Stabler M. (2020). Infectious Diseases as Socio-Spatial Processes: The COVID-19 Outbreak In Germany. Tijdschr. Econ. Soc. Geogr..

[9] Plümper T., Neumayer E. (2020). The pandemic predominantly hits poor neighbourhoods? SARS-CoV-2 infections and COVID-19 fatalities in German districts. Eur. J. Public Health.

[10] Wachtler B., Michalski N., Nowossadeck E., Diercke M., Wahrendorf M., Santos-Hövener C., Lampert T., Hoebel J. (2020). Sozioökonomische Ungleichheit im Infektionsrisiko mit SARS-CoV-2—Erste Ergebnisse einer Analyse der Meldedaten für Deutschland.

[11] Siljander M., Uusitalo R., Pellikka P., Isosomppi S., Vapalahti O. (2022). Spatiotemporal clustering patterns and sociodemographic determinants of COVID-19 (SARS-CoV-2) infections in Helsinki, Finland. Spat. Spatiotemporal. Epidemiol..

[12] Nazia N., Law J., Butt Z.A. (2022). Spatiotemporal clusters and the socioeconomic determinants of COVID-19 in Toronto neighbourhoods, Canada. Spat. Spatiotemporal. Epidemiol..

[13] Lu Y., Cai G., Hu Z., He F., Jiang Y., Aoyagi K. (2022). Exploring spatiotemporal patterns of COVID-19 infection in Nagasaki Prefecture in Japan using prospective space-time scan statistics from April 2020 to April 2022. Arch. Public Health.

[14] Iyanda A.E., Boakye K.A., Lu Y., Oppong J.R. (2022). Racial/Ethnic Heterogeneity and Rural-Urban Disparity of COVID-19 Case Fatality Ratio in the USA: A Negative Binomial and GIS-Based Analysis. J. Racial Ethn. Health Disparities.

[15] Lee J., Ramírez I.J. (2022). Geography of Disparity: Connecting COVID-19 Vulnerability and Social Determinants of Health in Colorado. Behav. Med..

[16] Rohleder S., Bozorgmehr K. (2021). Monitoring the spatiotemporal epidemiology of Covid-19 incidence and mortality: A small-area analysis in Germany. Spat. Spatiotemporal. Epidemiol..

[17] Adin A., Congdon P., Santafé G., Ugarte M.D. (2022). Identifying extreme COVID-19 mortality risks in English small areas: A disease cluster approach. Stoch. Environ. Res. Risk Assess..

[18] Dhewantara P.W., Puspita T., Marina R., Lasut D., Riandi M.U., Wahono T., Ridwan W., Ruliansyah A. (2022). Geo-clusters and socio-demographic profiles at village-level associated with COVID-19 incidence in the metropolitan city of Jakarta: An ecological study. Transbound. Emerg. Dis..

[19] Greene S.K., Peterson E.R., Balan D., Jones L., Culp G.M., Fine A.D., Kulldorff M. (2021). Detecting COVID-19 Clusters at High Spatiotemporal Resolution, New York City, New York, USA, June–July 2020. Emerg. Infect. Dis..

[20] Fatima M., O’Keefe K.J., Wei W., Arshad S., Gruebner O. (2021). Geospatial Analysis of COVID-19: A Scoping Review. Int. J. Environ. Res. Public Health.

[21] Booth A., Reed A.B., Ponzo S., Yassaee A., Aral M., Plans D., Labrique A., Mohan D. (2021). Population risk factors for severe disease and mortality in COVID-19: A global systematic review and meta-analysis. PLoS ONE.

[22] Sandoval M., Nguyen D.T., Vahidy F.S., Graviss E.A. (2021). Risk factors for severity of COVID-19 in hospital patients age 18–29 years. PLoS ONE.

[23] Meurisse M., Lajot A., Devleesschauwer B., van Cauteren D., van Oyen H., van den Borre L., Brondeel R. (2022). The association between area deprivation and COVID-19 incidence: A municipality-level spatio-temporal study in Belgium, 2020–2021. Arch. Public Health.

[24] Madhav K.C., Oral E., Straif-Bourgeois S., Rung A.L., Peters E.S. (2020). The effect of area deprivation on COVID-19 risk in Louisiana. PLoS ONE.

[25] Maier W., Fairburn J., Mielck A. (2012). Regionale Deprivation und Mortalität in Bayern. Entwicklung eines ’Index Multipler Deprivation’ auf Gemeindeebene. Gesundheitswesen.

[26] Kauhl B., Maier W., Schweikart J., Keste A., Moskwyn M. (2018). Who is where at risk for Chronic Obstructive Pulmonary Disease? A spatial epidemiological analysis of health insurance claims for COPD in Northeastern Germany. PLoS ONE.

[27] Odoi A., Busingye D. (2014). Neighborhood geographic disparities in heart attack and stroke mortality: Comparison of global and local modeling approaches. Spat. Spatiotemporal. Epidemiol..

[28] Lawson A.B., Biggeri A.B., Boehning D., Lesaffre E., Viel J.F., Clark A., Schlattmann P., Divino F. (2000). Disease mapping models: An empirical evaluation. Disease Mapping Collaborative Group. Stat. Med..

[29] Dwyer-Lindgren L., Cork M.A., Sligar A., Steuben K.M., Wilson K.F., Provost N.R., Mayala B.K., VanderHeide J.D., Collison M.L., Hall J.B. (2019). Mapping HIV prevalence in sub-Saharan Africa between 2000 and 2017. Nature.

[30] Kauhl B., Vietzke M., König J., Schönfelder M. (2022). Exploring regional and sociodemographic disparities associated with unenrollment for the disease management program for type 2 Diabetes Mellitus using Bayesian spatial modelling. Res. Health Serv. Reg.

[31] Lindgren F., Rue H. (2015). Bayesian Spatial Modelling with R—INLA. J. Stat. Soft..

[32] Wickham H., Winston C., Henry L., Lin Pedersen T. (2016). Package ‘ggplot2’. Create Elegant Data Visualisations using the Grammar of Graphics. Version 2.1. https://cran.r-project.org/package=ggplot2/ggplot2.pdf.

[33] Bland J.M., Altman D.G. (2000). Statistics notes. The odds ratio. BMJ.

[34] Anderson R.P., Jin R., Grunkemeier G.L. (2003). Understanding logistic regression analysis in clinical reports: An introduction. Ann. Thorac. Surg..

[35] Heiberger R.M. Package ‘HH’. Statistival Analysis and Data Display: Heidberger and Holland. https://cran.r-project.org/web/packages/HH/HH.pdf.

[36] Schüler L., Calabrese J.M., Attinger S. (2021). Data driven high resolution modeling and spatial analyses of the COVID-19 pandemic in Germany. PLoS ONE.

[37] Dragano N., Rupprecht C.J., Dortmann O., Scheider M., Wahrendorf M. (2021). Higher risk of COVID-19 hospitalization for unemployed: An analysis of health insurance data from 1.28 million insured individuals in Germany. Bundesgesundheitsblatt Gesundh. Gesundh..

[38] Mena G.E., Martinez P.P., Mahmud A.S., Marquet P.A., Buckee C.O., Santillana M. (2021). Socioeconomic status determines COVID-19 incidence and related mortality in Santiago, Chile. Science.

[39] Doblhammer G., Kreft D., Reinke C. (2021). Regional Characteristics of the Second Wave of SARS-CoV-2 Infections and COVID-19 Deaths in Germany. Int. J. Environ. Res. Public Health.

[40] Hayward S.E., Deal A., Cheng C., Crawshaw A., Orcutt M., Vandrevala T.F., Norredam M., Carballo M., Ciftci Y., Requena-Méndez A. (2021). Clinical outcomes and risk factors for COVID-19 among migrant populations in high-income countries: A systematic review. J. Migr. Health.

[41] Said D., Suwono B., Schweickert B., Schönfeld V., Eckmanns T., Haller S. (2022). SARS-CoV-2 Outbreaks in Care Homes for the Elderly and Disabled in Germany. Dtsch. Arztebl. Int..

[42] McGowan V.J., Bambra C. (2022). COVID-19 mortality and deprivation: Pandemic, syndemic, and endemic health inequalities. Lancet Public Health.

[43] Salkeld D.J., Antolin M.F. (2020). Ecological Fallacy and Aggregated Data: A Case Study of Fried Chicken Restaurants, Obesity and Lyme Disease. Ecohealth.

[44] Kauhl B., Schweikart J., Krafft T., Keste A., Moskwyn M. (2016). Do the risk factors for type 2 diabetes mellitus vary by location? A spatial analysis of health insurance claims in Northeastern Germany using kernel density estimation and geographically weighted regression. Int. J. Health. Geogr..

[45] Goffrier B., Schulz M., Bätzing-Feigenbaum J. Administrative Prevalence and Incidence of Diabetes Mellitus in Germany, 2009–2015. https://www.versorgungsatlas.de/fileadmin/ziva_docs/79/VA-79-Abstract_EN_Final.pdf.

[46] Akmatov M.K., Steffen A., Holstiege J., Bätzing J. (2019). Die Chronisch Obstruktive Lungenerkrankung (COPD) in der Ambulanten Versorgung in Deutschland–Zeitliche Trends und Kleinräumige Unterschiede. https://www.versorgungsatlas.de/fileadmin/ziva_docs/99/VA_19-06_Bericht-COPD_2019-08-20_V2_1.pdf.

